# Cerebrospinal Fluid Proenkephalin Predicts Striatal Atrophy Decades before Clinical Motor Diagnosis in Huntington's Disease

**DOI:** 10.1002/mds.70062

**Published:** 2025-09-26

**Authors:** Mena Farag, Michael J. Murphy, Nicola Z. Hobbs, Michela Leocadi, Kate Fayer, Olivia Thackeray, Johan Gobom, Marc Ciosi, Amanda Heslegrave, Henrik Zetterberg, Douglas R. Langbehn, Darren G. Monckton, Edward J. Wild, Sarah J. Tabrizi, Rachael I. Scahill

**Affiliations:** ^1^ Huntington's Disease Centre, Department of Neurodegenerative Disease, UCL Queen Square Institute of Neurology University College London London United Kingdom; ^2^ Department of Psychiatry and Neurochemistry, Institute of Neuroscience and Physiology, Sahlgrenska Academy at University of Gothenburg Mölndal Sweden; ^3^ Clinical Neurochemistry Laboratory Sahlgrenska University Hospital Mölndal Sweden; ^4^ School of Molecular Biosciences, College of Medical, Veterinary and Life Sciences University of Glasgow Glasgow United Kingdom; ^5^ Dementia Research Institute University College London London United Kingdom; ^6^ Department of Neurodegenerative Disease UCL Queen Square Institute of Neurology London United Kingdom; ^7^ Hong Kong Center for Neurodegenerative Diseases Clear Water Bay Hong Kong China; ^8^ Wisconsin Alzheimer's Disease Research Center, University of Wisconsin School of Medicine and Public Health University of Wisconsin‐Madison Madison Wisconsin USA; ^9^ Centre for Brain Research Indian Institute of Science Bangalore India; ^10^ Department of Psychiatry and Biostatistics, Carver College of Medicine and College of Public Health University of Iowa Iowa City Iowa USA

**Keywords:** Huntington's disease; proenkephalin; striatal atrophy; voxel‐based morphometry

## Abstract

**Background:**

Huntington's disease (HD) is characterized by early, selective, progressive vulnerability of striatal medium spiny neurons (MSNs). Proenkephalin (PENK), a precursor of opioid peptides abundantly expressed in MSNs, is a promising biomarker of striatal integrity, but region‐specific associations and its potential for early‐stage discrimination have not been characterized.

**Objectives:**

We investigated cross‐sectional and longitudinal associations between baseline cerebrospinal fluid (CSF) PENK concentration and regional brain atrophy, compared identified patterns with CSF neurofilament light (NfL), and evaluated PENK and NfL for discriminating between HD Integrated Staging System (HD‐ISS) stage 0 versus 1 in a far‐from‐onset HD gene‐expanded (HDGE) cohort.

**Methods:**

Whole‐brain voxel‐based morphometry was performed in 149 participants (72 HDGE, 77 controls) cross‐sectionally and 88 participants (54 HDGE, 34 controls) longitudinally over a mean interval of 4.8 years. Voxel‐wise linear regression tested associations between baseline biofluid biomarkers and gray/white matter volume, adjusting for age, sex and CAG‐Age Product score, with false discovery rate correction. Logistic regression and receiver operating characteristic analyses assessed stage discrimination.

**Results:**

Lower baseline CSF PENK predicted longitudinal gray and white matter loss, predominantly in the striatum bilaterally. Higher baseline CSF NfL predicted widespread longitudinal white matter loss. For stage discrimination, PENK (area under curve [AUC], 0.706; *P* = 0.0002) outperformed NfL (AUC, 0.661; *P* = 0.1596) with minimal gain from combining both (AUC, 0.714; joint *P* = 0.0007).

**Conclusions:**

Lower baseline CSF PENK concentration predicted longitudinal striatal atrophy and CSF PENK outperformed CSF NfL in distinguishing HD‐ISS stages 0 and 1, supporting its role as a striatum‐specific biomarker with potential to enrich early‐stage HD trial cohorts. © 2025 The Author(s). *Movement Disorders* published by Wiley Periodicals LLC on behalf of International Parkinson and Movement Disorder Society.

Huntington's disease (HD) is an autosomal dominant neurodegenerative disorder caused by a cytosine‐adenine‐guanine (CAG) trinucleotide repeat expansion in exon 1 of the *HTT* gene, producing an abnormally long polyglutamine tract in the huntingtin (HTT) protein.^1^ Expansions of ≥40 CAGs are fully penetrant, invariably leading to disease within an affected individual's lifetime. The adult‐onset phenotype is typically characterized by a triad of motor, cognitive and neuropsychiatric symptoms, with a median survival of approximately 20 years from clinical motor onset.^2^ Despite advances in the therapeutic landscape since the discovery of *HTT* in 1993,^3^, ^4^ no disease‐modifying treatments are currently available.^5^ Delineating the earliest pathological changes in HD are critical to inform the design of future preventive trials and interventions in presymptomatic HD gene‐expanded (HDGE) cohorts.

The neuropathological hallmark of HD is a progressive and regionally‐selective pattern of cellular degeneration, most prominently affecting the striatum within the basal ganglia.^6^ Among the most susceptible and vulnerable cell populations are striatal medium spiny neurons (MSNs).^7^, ^8^, ^9^ Striatal MSNs contribute to the direct and indirect pathways of the basal ganglia and respond to dopaminergic signaling in part via endogenous opioid systems.^10^, ^11^ These neurons are predominantly inhibitory, using γ‐aminobutyric acid as their principal neurotransmitter and co‐express neuropeptides that act on opioid receptors.^11^, ^12^ Proenkephalin (PENK), an opioid peptide precursor, is enriched in striatal MSNs of the indirect pathway projecting to the globus pallidus externus.^13^, ^14^ In the rat brain, the caudate and putamen are densely populated in enkephalin‐containing neurons expressing high levels of prePENK mRNA.^15^, ^16^ In humans, PENK mRNA is most highly expressed in the basal ganglia, followed by the adrenal gland and testis.^17^ Postmortem brain tissue from people with HD demonstrated reduced methionine‐enkephalin content across basal ganglia nuclei compared to controls,^18^ supporting the hypothesis that enkephalin‐containing neurons are among the selectively vulnerable neuronal populations affected in HD.

We recently demonstrated in the HD Young Adult Study (HD‐YAS) that decades before predicted clinical motor diagnosis, HDGE participants, despite preserved clinical function, show subtle yet significant volumetric brain changes over approximately 4.5 years, compared with matched controls.^19^ In parallel, cerebrospinal fluid (CSF) concentration of neurofilament light (NfL; a marker of neuroaxonal injury) was significantly elevated, whereas PENK concentration was significantly reduced over time in the HDGE cohort, compared to controls.

In cross‐sectional studies, CSF PENK concentration has been found to be significantly reduced in manifest HD cohorts compared to controls,^20^, ^21^ with high discriminative power,^22^ and showing specificity for HD compared other neurodegenerative conditions.^23^ In our HD‐YAS cohort, baseline CSF PENK concentration was a robust predictor of subsequent brain atrophy, most significantly within the caudate and putamen, independent of age and CAG repeat length.^19^ Importantly, these findings support our hypothesis that CSF PENK is a promising biomarker of striatal MSN integrity, with declining concentration reflecting progressive striatal atrophy and/or dysfunction. Despite this promise, the potential of PENK as a selective biomarker of striatal damage in HD has not yet been studied using regional brain imaging.

Johnson et al^24^ previously used whole‐brain voxel‐based morphometry (VBM)^25^ to determine how plasma NfL concentration predicted regional volume loss in HD, which is principally cortical gray and subcortical white matter, perhaps unsurprisingly for a structural component of axons. In this study, we investigated both cross‐sectional and longitudinal associations between baseline CSF PENK concentration and regionally‐specific neurodegeneration in the far‐from‐onset HD‐YAS HDGE cohort.^19^, ^26^ We hypothesized that lower baseline CSF PENK concentration would be associated with regionally‐specific brain atrophy focused around the striatum. Additionally, we sought to compare the regional patterns of PENK‐related structural associations and changes with those of other fluid biomarkers that showed disease‐related group differences. As a further aim, we also assessed the ability of CSF PENK and CSF NfL to discriminate between HD Integrated Staging System (HD‐ISS)^27^ stages 0 and 1, individually and in combination, to investigate their potential utility as surrogate markers of early disease staging.

## Subjects and Methods

### Study Cohort

Participants were enrolled from the single‐site longitudinal HD‐YAS.^19^, ^26^ At baseline, 131 participants were enrolled (64 HDGE participants, 67 controls).^26^ At approximately 4.5 year follow‐up, 103 participants returned (57 HDGE participants, 46 controls) and 23 new participants were recruited (nine HDGE participants, 14 controls).^19^

Inclusion criteria for the HDGE cohort were age 18 to 40 years, CAG repeat length ≥40 and Disease Burden Score (DBS) ≤240 (DBS = age × [CAG‐35.5]), corresponding to approximately two decades before predicted clinical motor diagnosis.^28^, ^29^ Full study design, recruitment, and HD‐ISS staging procedures have been reported previously.^19^

### Standard Protocol Approvals, Registrations, and Patient Consents

The study was registered on ClinicalTrials.gov (NCT06391619), where the full protocol and pre‐specified statistical analysis plan are available. Ethical approval was granted by the London–Bloomsbury Research Ethics Committee (22/LO/0058). All participants provided written informed consent before enrolment.

### Quantification of Fluid Biomarkers

CSF and plasma samples were collected at baseline and follow‐up under identical, standardized protocols using validated methods and equipment.^30^ CSF PENK concentrations were quantified by liquid chromatography‐mass spectrometry with tandem mass tag multiplexing.^19^, ^31^ CSF and plasma NfL concentrations were measured using the Neurology 4‐Plex A assay on the Simoa HD‐X platform (Quanterix Corp., Billerica, MA, USA). CSF chitinase‐3‐like protein 1 (YKL‐40) concentrations were measured using the Human YKL‐40 assay on the U‐PLEX platform (Meso Scale Discovery (MSD), Rockville, MD, USA).

### Imaging Acquisition and Processing

Structural magnetic resonance imaging (MRI) was performed on a 3‐Tesla Prisma scanner (Siemens Healthineers, Erlangen, Germany) using a three‐dimensional (3D) T1‐weighted magnetization prepared rapid gradient echo (MPRAGE) sequence (repetition time: 2530 ms, echo time: 3.34 ms, inversion time: 1100 ms, flip angle: 7°, field of view: 256 × 256 × 176 mm^3^, voxel size: 1.0 mm^3^). Acquisition parameters were identical at baseline and follow‐up, with no hardware or major software changes. To minimize temporal bias, baseline, and follow‐up scans were reprocessed together using identical pipelines.^19^

All T1‐weighted scans underwent quality control through visual inspection, blinded to group status. Baseline images were pre‐processed using the Computational Anatomy Toolbox (CAT12)^32^ within SPM12 (https://www.fil.ion.ucl.ac.uk/spm/software/spm12/), implemented in MATLAB R2022b (https://uk.mathworks.com), generating gray matter, white matter and CSF segmentations in native space, which were normalized to a study‐specific template created using diffeomorphic anatomical registration through exponentiated lie algebra (DARTEL).^33^ Normalized tissue maps were modulated to preserve volume and smoothed using an 8 mm full‐width at half‐maximum Gaussian kernel. Explicit masking was applied using a majority tissue mask to constrain analyses to voxels with a minimum tissue probability of 0.1 present in at least 70% of the cohort.^34^

Longitudinal VBM analysis was performed using established methods.^24^, ^35^, ^36^ Within‐subject structural change over time was quantified using a nonlinear fluid registration^37^ within the MIDAS software.^38^ For each participant, voxel‐compression maps representing local volume change from baseline to follow‐up were generated. These maps were then spatially normalized to the study‐specific DARTEL template and convolved with participant‐specific baseline gray and white matter segmentations, producing voxel‐wise estimates of within‐subject tissue change for gray and white matter.^35^ Intracranial volume (ICV) was measured using a semi‐automated procedure within the MIDAS platform.^38^, ^39^

### Statistical Analysis

Whole‐brain voxel‐wise analyses were performed using linear regression models within SPM12 to assess associations between structural brain changes and biofluid biomarker levels. Specifically, correlations were tested between baseline concentrations of PENK in CSF, NfL in CSF and plasma and YKL‐40 in CSF with (1) cross‐sectional baseline gray and white matter volume and (2) longitudinal changes in gray and white matter volume. All analyses used explicit tissue‐specific masks (gray or white matter).

Group differences in cross‐sectional gray and white matter volume and longitudinal atrophy in these tissues were conducted using two‐sample *t*‐tests within the SPM12 factorial design specification framework, covarying for age and sex. For cross‐sectional models, additional adjustment was made for ICV to account for inter‐individual variation in head size and in longitudinal models scan interval was included as a covariate.

To test associations between baseline biofluid biomarker levels and both cross‐sectional volume and longitudinal atrophy within the HDGE cohort, voxel‐wise linear regression was used, covarying for age, sex, MiSeq CAG‐Age Product (CAP) score and, only for longitudinal associations, the time interval between MRI acquisitions. The CAP100 score, with 100 corresponding to the CAG‐specific estimated age at clinical motor diagnosis,^40^ was included to account for cumulative disease burden to isolate the independent associations between biofluid biomarker and changes in gray and white matter volumes. Statistical significance was thresholded at a 5% false discovery rate (FDR) to correct for multiple comparisons. Clinical and demographic data were summarized descriptively. Group comparisons used χ^2^ tests for categorical and two‐sample *t*‐tests for continuous variables.

To assess the ability of CSF PENK and CSF NfL to discriminate between HD‐ISS stage 0 versus 1 in the HDGE cohort, logistic regression models were fitted with HD‐ISS stage (stage 1 vs. stage 0) as the dependent variable, and log transformed CSF PENK or CSF NfL values as the primary independent variable, adjusting for age, sex and CAP100 score. HDGE participants with paired imaging‐derived HD‐ISS stage and CSF sample from the same visit were included (n = 113). Models were estimated with robust standard errors clustered by participant to account for repeated measures. Predicted probabilities from each model were used to generate receiver operating characteristic (ROC) curves and calculate the area under the curve (AUC) with 95% confidence intervals (CI). The analysis was repeated with both biomarkers entered simultaneously to assess incremental predictive value. Wald χ^2^ tests were used to assess the significance of each biomarker individually, and a joint test was used to assess the combined contribution of both biomarkers. All analyses were performed in Stata MP, version 18.0 (StataCorp, College Station, TX, USA).

## Results

### Cross‐Sectional Analyses

A total of 149 cross‐sectional MRI scans were available for VBM analyses, comprising 72 HDGE participants and 77 controls (Table S1). Cross‐sectional comparisons of gray and white matter volume revealed no significant group differences.

HDGE participants were included in the cross‐sectional VBM‐biofluid association analyses if they had an MRI scan acquired at either the baseline or follow‐up visit and available associated biofluid samples. Of the 72 HDGE participants who met imaging criteria, six were excluded because of missing associated biofluid data, leaving 66 participants (Table 1). At the time of the cross‐sectional scan, 49 participants (74%) were in HD‐ISS stage 0, 16 (24%) in stage 1 and one (2%) in stage 2. Within this HDGE cohort, there were no significant associations between baseline concentrations of CSF PENK and YKL‐40, or CSF and plasma NfL and cross‐sectional gray or white matter volume.

**TABLE 1:** Characteristics of the cross‐sectional and longitudinal HDGE cohort included in the VBM‐biofluid analyses

Characteristic	Cross‐sectional	Longitudinal	
No. of HDGE participants	66	50	
Sex, n (%): female:male	35:31 (53:47)	24:26 (48:52)	
Age at baseline (years)	30.0 (SD, 5.5)	29.9 (SD, 5.6)	
Interval (years)	N/A	4.9 (SD, 0.6)	
HD‐ISS	Stage (at time of scan):	Visit 1	Visit 2
Stage 0, n (%)	49 (74)	41 (82)	32 (64)
Stage 1, n (%)	16 (24)	8 (16)	17 (34)
Stage 2, n (%)	1 (2)	1 (2)	1 (2)
MiSeq CAG repeat length	42.2 (SD, 1.5)	42.3 (SD, 1.4)	
MiSeq CAP100 score at baseline	55.6 (SD, 8.2)	55.9 (SD, 8.6)	
DBS at baseline	196.2 (SD, 36.3)	198.1 (SD, 37.4)	
Estimated years to clinical motor diagnosis	22.3 (SD, 5.2)	22.1 (SD, 5.2)	
ICV (mL)	1502.5 (SD, 153.9)	N/A	
Baseline CSF PENK^a^	1.10 (IQR, 1.00–1.30)	1.10 (IQR, 0.90–1.30)	
Baseline CSF NfL (pg/mL)	413 (IQR, 270–652)	427 (IQR, 310–680)	
Baseline plasma NfL (pg/mL)	8.50 (IQR, 6.30–11.2)	8.90 (IQR, 7.10–11.2)	
Baseline CSF YKL‐40 (pg/mL)	82,200 (IQR, 66,600–101,000)	84,300 (68,400–105,000)	

*Note*: Values are n (%), mean (SD) or median (IQR), as appropriate.

^a^ Relative intensity (TMT ratio, log‐transformed).

Abbreviations: HDGE, Huntington's disease gene‐expanded; VBM, voxel‐based morphometry; SD, standard deviation; HD‐ISS, HD Integrated Staging System; CAG, cytosine‐adenine‐guanine; CAP100, CAG‐Age Product score scaled to 100; DBS, Disease Burden Score; ICV, intracranial volume; CSF, cerebrospinal fluid; PENK, proenkephalin; IQR, interquartile range; NfL, neurofilament light; YKL‐40, chitinase‐3‐like protein 1; TMT, tandem mass tag.

### Longitudinal Analyses

A total of 88 longitudinal MRI scans were available for VBM analyses, comprising 54 HDGE participants and 34 controls (Table S2). Of the 64 HDGE participants assessed at baseline,^26^ seven dropped out and did not complete follow‐up. The dropout group had a mean (standard deviation [SD]) MiSeq CAG repeat length of 42.7 (2.4), mean age of 27.6 (6.2) years, and mean CAP100 score of 52.9 (9.5); five were female and two male; and four were in HD‐ISS stage 1 and three in stage 0. Although excluded from longitudinal analyses, six of the seven dropouts (all except one without CSF sampling) were included in cross‐sectional analyses. In addition, three further participants were scanned only once: one was not scanned at baseline because of claustrophobia but completed the follow‐up scan, one was unable to complete the follow‐up scan because of claustrophobia, and one was not scanned at follow‐up because of MRI safety concerns.

**FIG. 1:**
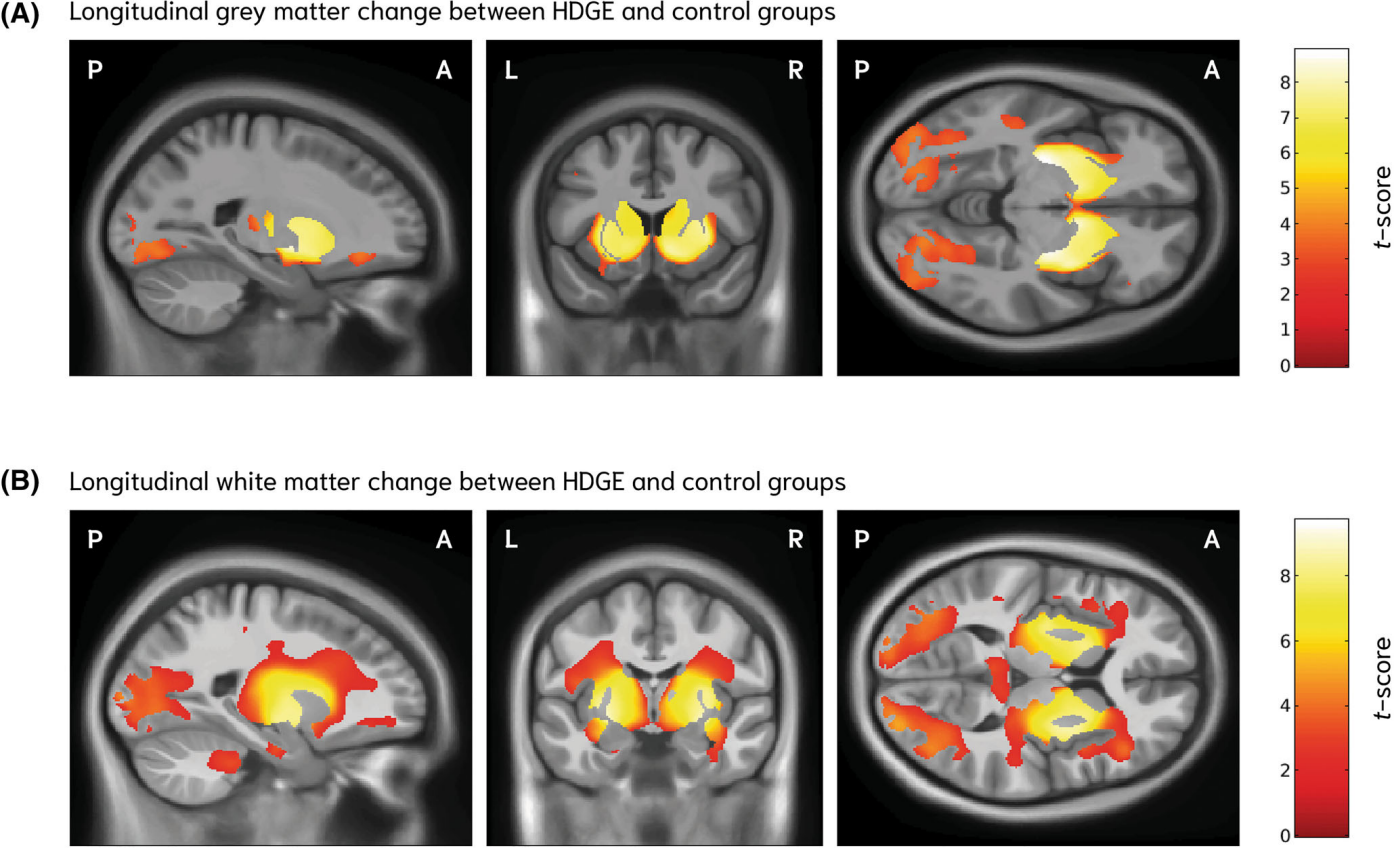
Group differences in longitudinal gray and white matter volume change. Statistical parametric maps showing differences in gray (A) and white (B) matter volume change over a mean follow‐up of 4.8 years between the Huntington's disease gene‐expanded (HDGE) cohort (n = 54) and unaffected controls (n = 34). Compared with controls, the HDGE cohort showed symmetrical gray matter volume loss, most prominently in the bilateral caudate and putamen, with additional loss in the occipital cortex (A). White matter volume loss was greater in the HDGE cohort, centered on the striatum and extending into the parietal and temporal lobes, posterior regions, and cerebellar tracts (B). Statistical parametric maps are displayed on a study‐specific T1‐weighted group template in sagittal, coronal and axial planes. Orientation labels indicate P, posterior; A, anterior; L, left; and R, right. Color bars indicate t‐scores, with red denoting lower values and yellow to white denoting higher values, corresponding to greater group differences. Results are thresholded at 5% false discovery rate (FDR). All models were adjusted for age, sex, CAP100 score and the time interval between magnetic resonance imaging acquisitions.

Longitudinal volumetric analyses over a mean interval of 4.8 years revealed symmetrical gray matter volume loss in the HDGE cohort, most prominently in the caudate and putamen bilaterally (FDR *q* < 0.0001), with additional gray matter volume loss in the bilateral occipital cortices (FDR *q* < 0.004) and a small region in the left temporal lobe (FDR *q* < 0.02) (Fig. 1A; Table S3). Compared with controls, the HDGE cohort also showed greater white matter volume loss most prominently in the bilateral peristriatal white matter and occipital lobes (FDR *q* < 0.0001) (Fig. 1B; Table S3).

For the longitudinal VBM‐biofluid association analyses, of the 54 HDGE participants with longitudinal imaging, four were excluded because of missing baseline biofluid data, leaving 50 HDGE participants with longitudinal imaging over a mean interval of 4.9 years. At baseline 41 participants (82%) were in HD‐ISS stage 0, eight (16%) in stage 1 and one (2%) in stage 2. At follow‐up, nine participants had progressed from HD‐ISS stage 0 to 1, resulting in 32 (64%) in stage 0, 17 (34%) in stage 1 and one (2%) in stage 2. Baseline clinical characteristics and demographics of the HDGE cohort included in the longitudinal VBM analyses are summarized in Table 1.

### PENK and NfL both Predict Subsequent Atrophy, but PENK Is Striatally Selective

**FIG. 2:**
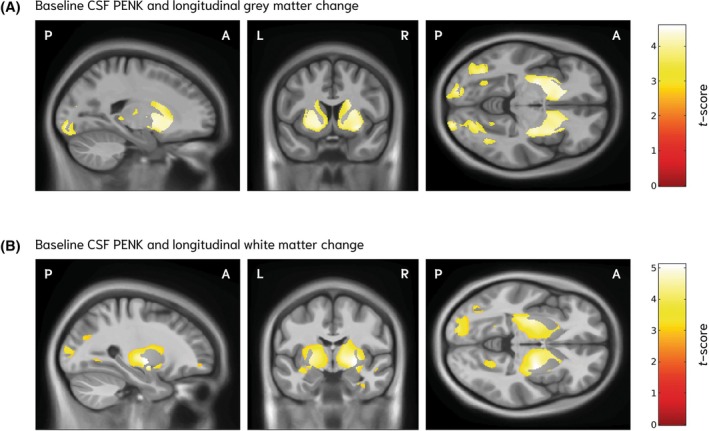
Associations between baseline baseline cerebrospinal fluid (CSF) proenkephalin (PENK) concentration and longitudinal gray and white matter volume change in the Huntington's disease gene‐expanded (HDGE) cohort. Voxel‐based morphometry results for the association between baseline CSF PENK concentration and longitudinal gray (A) and white (B) matter volume change in HDGE participants (n = 50) over a mean follow‐up of 4.9 years. Lower baseline PENK concentrations were associated with greater gray matter volume loss, primarily in the bilateral caudate and putamen, with additional occipital cortex involvement (A). Comparable significant associations were observed for white matter volume loss, centered on the bilateral striatum and extending into posterior and occipital white matter regions (B). Statistical parametric maps are displayed on a study‐specific T1‐weighted group template in sagittal, coronal and axial planes. Orientation labels indicate P, posterior; A, anterior; L, left; and R, right. Color bars indicate t‐scores, with red denoting lower values and yellow to white denoting higher values, corresponding to stronger associations. Results are thresholded at 5% false discovery rate (FDR). All models were adjusted for age, sex, CAP100 score and the time interval between magnetic resonance imaging acquisitions.

Baseline CSF PENK showed a prominent and selective association with subsequent striatal atrophy. Lower baseline CSF concentration of PENK was significantly associated with greater longitudinal gray matter volume loss, most prominently in the caudate and putamen bilaterally (FDR *q* < 0.014), with additional clusters in the occipital lobes (FDR *q* < 0.014) and the right temporal lobe (FDR *q* < 0.021) (Fig. 2A; Table S4). Similar associations were observed for white matter volume loss, centered on the peristriatal white matter (FDR *q* < 0.009) and extending into the white matter of the left occipital lobe (FDR *q* < 0.01) and right temporal lobe (FDR *q* < 0.03) (Fig. 2B; Table S4).

**FIG. 3:**
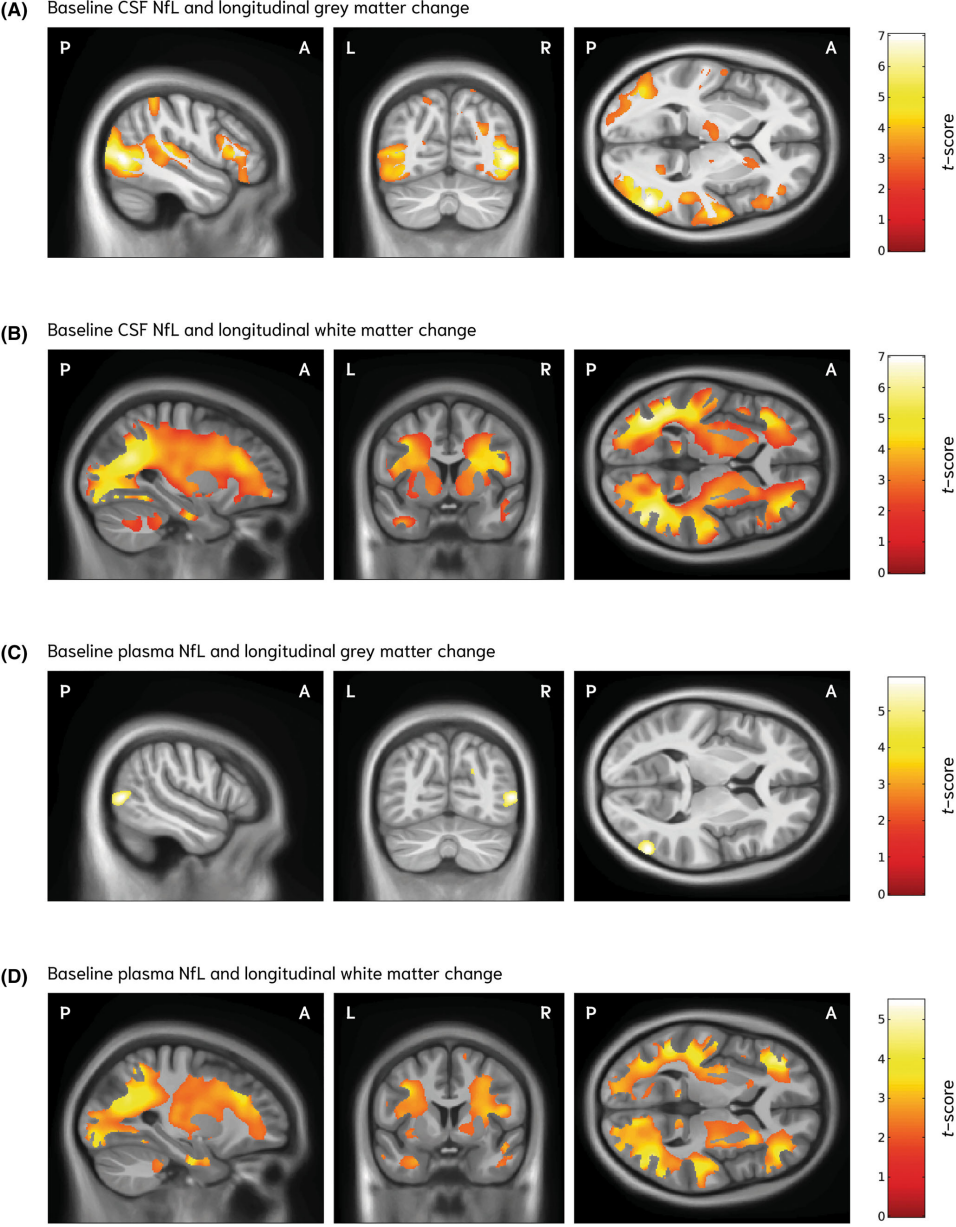
Associations between baseline neurofilament light (NfL) concentration and longitudinal gray and white matter volume change in a Huntington's disease gene‐expanded (HDGE) cohort. Voxel‐based morphometry results for the association between baseline baseline cerebrospinal fluid (CSF) NfL (A,B) and plasma NfL (C,D) concentrations and longitudinal gray and white matter volume change in HDGE participants (n = 50) over a mean follow‐up of 4.9 years. CSF NfL: higher baseline concentrations were associated with greater gray matter volume loss, most prominent in the bilateral occipital cortex with additional parietal and temporal involvement (A), and with widespread white matter volume loss affecting bilateral occipital, parietal and temporal white matter tracts (B). Plasma NfL: higher baseline concentrations were associated with limited gray matter volume loss, with small clusters in the right lateral occipital cortex (C), and with widespread white matter volume loss involving bilateral occipital, parietal, temporal and posterior frontal tracts (D). Statistical parametric maps are displayed on a study‐specific T1‐weighted group template in sagittal, coronal and axial planes. Orientation labels indicate P, posterior; A, anterior; L, left; and R, right. Color bars indicate t‐scores, with red denoting lower values and yellow to white denoting higher values, corresponding to stronger associations. Results are thresholded at 5% false discovery rate (FDR). All models were adjusted for age, sex, CAP100 score and the time interval between magnetic resonance imaging acquisitions.

In contrast to PENK, baseline CSF NfL predicted subsequent atrophy more globally with a preponderance for cortical gray and subcortical white matter. Higher baseline CSF NfL concentration was significantly associated with greater longitudinal gray matter volume loss in the bilateral occipital cortices (FDR *q* < 0.001) with additional involvement of bilateral temporo‐parietal regions (FDR *q* < 0.04) and the right frontal lobe (FDR *q* < 0.02) (Fig. 3A; Table S5). Subcortical involvement was limited to the right striatum (FDR *q* < 0.03) and bilateral thalamus (FDR *q* < 0.04). For longitudinal white matter volume changes, higher baseline CSF NfL was significantly associated with widespread atrophy across occipital, parietal, temporal and frontal lobes, and subcortical white matter (FDR <0.001) and bilaterally in the cerebellum (FDR *q* < 0.03) (Fig. 3B; Table S5).

Compared to CSF NfL, baseline concentrations of NfL in plasma was associated with a more restricted pattern of gray matter volume loss. Gray matter associations were limited and asymmetrical, with small clusters localized to the right lateral occipital gyrus (FDR *q* < 0.02) (Fig. 3C; Table S6). In contrast, higher baseline plasma NfL concentration was significantly associated with widespread longitudinal white matter volume loss, particularly in occipital, parietal, temporal and frontal lobes, and subcortical white matter (FDR *q* < 0.008) (Fig. 3D; Table S6).

There were no significant associations between baseline CSF YKL‐40 and longitudinal gray or white matter change at a threshold of 5% FDR.

### CSF PENK versus NfL for Discrimination of HD‐ISS Stage 0 versus Stage 1

**FIG. 4:**
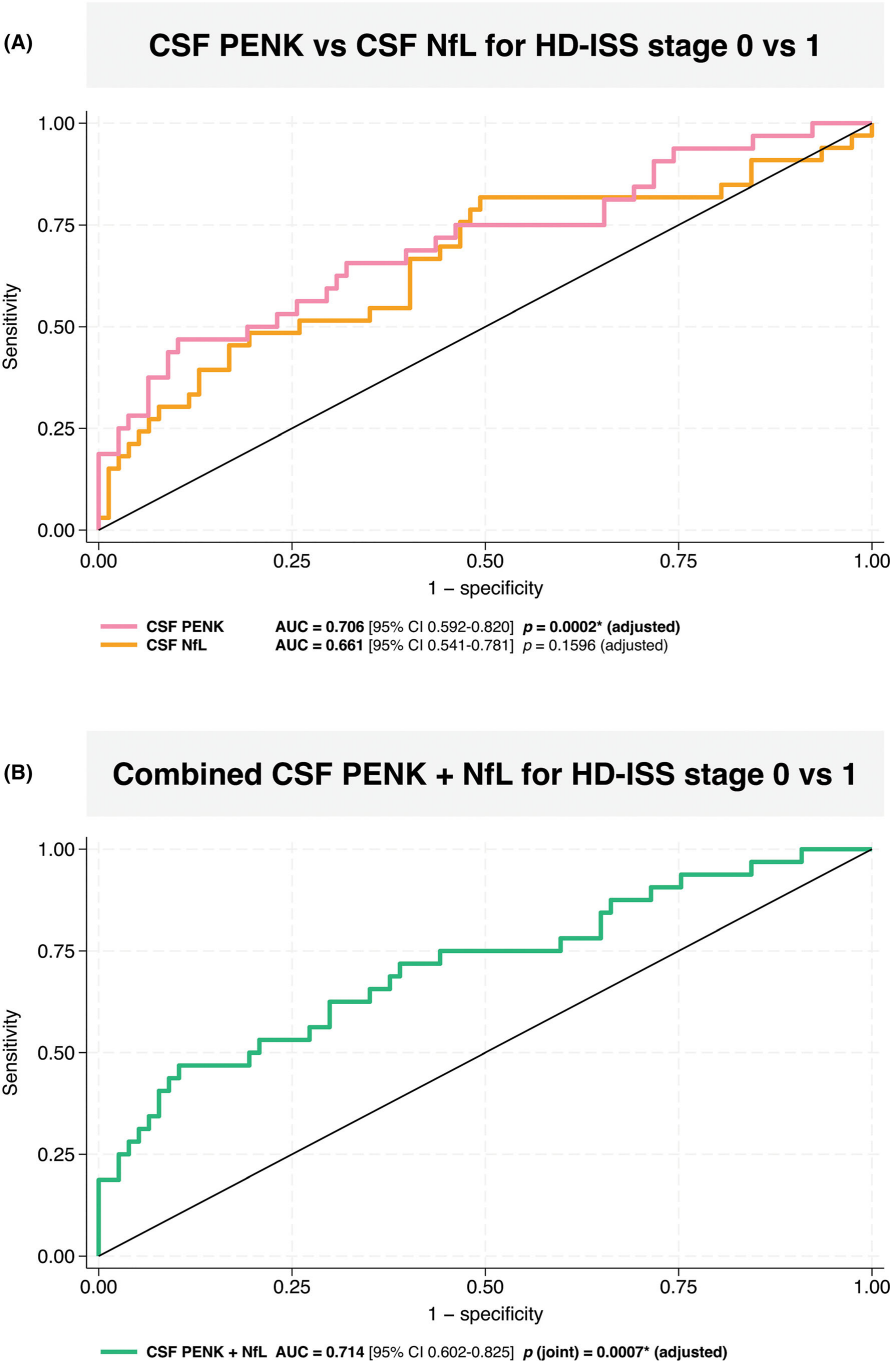
Receiver operating characteristic (ROC) curves for cerebrospinal fluid (CSF) proenkephalin (PENK) and CSF neurofilament light (NfL) in discriminating Huntington's disease Integrated Staging System (HD‐ISS) stage 1 from stage 0. (A) ROC curves for CSF PENK (pink) and CSF NfL (orange) from logistic regression models adjusted for age, sex and CAP100 score. CSF PENK showed higher discrimination (area under curve [AUC], 0.706; 95% confidence interval [CI], 0.592–0.820; *P* = 0.0002) than CSF NfL (AUC, 0.661; 95% CI, 0.541–0.781; *P* = 0.1596).(B) ROC curve for the combined model (green) including both CSF PENK and CSF NfL, adjusted for age, sex and CAP100 (AUC, 0.714; 95% CI, 0.602–0.825; joint test *P* = 0.0007). Diagonal line indicates chance discrimination (AUC, 0.5).

We assessed the ability of CSF PENK and CSF NfL to distinguish between HD‐ISS stages 0 and 1. In logistic regression models adjusted for age, sex, and CAP100, CSF PENK was strongly associated with HD‐ISS stage 1 status (*P* = 0.0002), whereas CSF NfL was not (*P* = 0.1596). Corresponding ROC analyses showed an AUC of 0.706 (95% CI, 0.592–0.820) for PENK and 0.661 (95% CI, 0.541–0.781) for NfL (Fig. 4A). Inclusion of both biomarkers in the same adjusted model yielded a combined AUC of 0.714 (95% CI, 0.602–0.825) (Fig. 4B), a modest increase over PENK alone. In this model, PENK remained an independent predictor (*P* = 0.001), whereas NfL did not contribute additional predictive value (*P* = 0.854). The joint test for both biomarkers was highly significant (*P* = 0.0007), indicating that the combined model discriminated HD‐ISS stage 0 from stage 1 better than chance.

## Discussion

This study presents spatially unbiased, whole‐brain voxel‐wise analyses showing, for the first time, that lower baseline CSF PENK concentration is a significant predictor of longitudinal reductions in gray and white matter volumes mainly within the striatum in a far‐from‐onset HDGE cohort. Beyond its strong regional specificity for striatal degeneration, CSF PENK demonstrated superior ability to discriminate between HD‐ISS stage 0 and stage 1 compared with CSF NfL, with minimal incremental benefit from combining the two biomarkers. The findings from this study provide robust evidence for CSF PENK as a strikingly striatum‐specific biomarker with sensitivity decades from predicted clinical motor diagnosis.

This stage‐discrimination ability of CSF PENK could support enrichment of very early trial cohorts by identifying HD‐ISS stage 0 to 1 progressors, the latter defined by striatal volumes below age‐adjusted norms.^27^ Although current HD‐ISS criteria do not formally include wet biomarkers,^27^ the integration of robust wet biomarkers such as CSF PENK and NfL could enrich the staging framework and potentially facilitate biomarker‐based classification within stages 0 and 1. Our findings indicate that CSF PENK is unlikely to replace CSF NfL, because the two reflect distinct pathological processes: PENK signals striatal MSN injury or loss, whereas NfL reflects more generalized axonal injury. PENK may, therefore, be most valuable alongside NfL, particularly for enriching HD‐ISS stage 0 or 1 clinical trial populations or monitoring striatal‐specific injury.

Baseline CSF PENK concentration predicted subsequent longitudinal brain volume loss, predominantly in the striatum, over a mean interval of 4.9 years in this far‐from‐onset cohort. These associations remained significant even after adjusting for the effects of CAG repeat length and CAP100 score, both well‐established predictors of HD progression,^41^, ^42^, ^43^ indicating an independent relationship with bilateral striatal gray and white matter loss. The additional observed association with clusters in the occipital cortex may reflect degeneration of striatal‐motor‐occipital connectivity, previously characterized using diffusion tractography.^44^ Given that the caudate tail, an early site of neurodegeneration in HD, receives projections from the occipital cortex,^45^, ^46^ these findings may also reflect disruption along this visual corticostriatal pathway.

Although PENK appears to be more specific to striatal pathology, NfL predominantly reflects associations with widespread white matter changes. This is consistent with the established role of NfL as a non‐specific marker of neuroaxonal injury, being a protein highly expressed in large‐caliber myelinated axons.^47^ Our finding that baseline CSF NfL concentration predicts widespread longitudinal white matter volume reduction, along with gray matter volume loss predominantly affecting the bilateral occipital cortex and, to a lesser extent, parietal and temporal regions, is consistent with previous VBM analyses from the TRACK‐HD cohort.^24^ In that study, higher baseline plasma NfL concentration predicted progressive gray and white matter atrophy over 3 years.^24^ Notably, the TRACK‐HD cohort was much closer to predicted clinical motor diagnosis (median, 10.8 years from onset at baseline),^48^ compared with the HD‐YAS cohort in this study where the HDGE cohort had an estimated mean of 22.1 years from predicted clinical motor diagnosis. Although higher baseline plasma NfL concentration was also associated with widespread white matter volume reductions, gray matter associations with plasma concentrations in our study were limited to small clusters primarily localized to the right lateral occipital cortex. This may reflect the lower sensitivity of plasma NfL compared with CSF NfL at this very early stage of disease, supported by earlier findings that at baseline, only 53% of HDGE participants had CSF NfL concentrations within the normal range, compared to 87% within the 95^th^ percentile for plasma NfL.^26^ Nevertheless, the occipital lobe is recognized as one of the earliest cortical regions to undergo atrophy in HD.^49^, ^50^

The absence of significant associations between baseline CSF YKL‐40 concentration and longitudinal gray or white matter volume change may reflect its role as a diffuse marker of astrocytic activation without regional specificity. Cross‐sectional analyses revealed no significant group differences in gray or white matter volumes and no associations with baseline biofluid biomarker concentrations, consistent with baseline HD‐YAS findings.^51^

Despite the increase in sample size, cross‐sectional differences between HDGE and controls remained non‐significant. The absence of cross‐sectional differences likely reflects the homogeneity of this very early‐stage cohort, in whom disease‐related brain changes are still subtle, unlike later cohorts such as TRACK‐HD,^48^ where cross‐sectional comparisons reflect the accumulation of neurodegeneration over many more years. These findings suggest that, although cross‐sectional group differences are not yet detectable, early pathological processes are underway and have begun to accelerate in this cohort, since the 4.9‐year interval showed more detectable change. It remains to be seen what the shortest interval is for detecting biomarker and imaging change in far‐from‐onset HDGE cohorts.

Regarding study limitations, all analyses were based on the recruitment of highly motivated participants to a single site, which may limit the generalizability of our findings. Additionally, the whole‐brain, voxel‐wise approach, although unbiased, reduces statistical power compared to region‐of‐interest analyses. Although we identified significant longitudinal associations, future studies in larger, independent cohorts may have increased the power to detect cross‐sectional effects. A small number of participants (n = 7) did not complete longitudinal follow‐up, however, six dropouts were still included in the cross‐sectional analyses, and the low dropout rate is unlikely to have influenced the main findings.

Although our follow‐up interval does not capture short‐term dynamics of biofluid biomarker changes, baseline CSF PENK strongly predicted subsequent striatal atrophy, supporting its prognostic potential. More frequent CSF sampling will be needed to determine whether PENK changes quickly enough to detect treatment effects over the shorter intervals typical of early interventional trials. At present, its greatest utility is likely at the group level, with individual‐level application contingent on defining reliable change thresholds in larger datasets. Studies in independent cohorts are needed to further characterize the distribution and longitudinal trajectory of CSF PENK across HD‐ISS stages 0 and 1, establish thresholds predictive of stage progression and evaluate its performance in combination with other biofluid biomarkers. Such work will clarify whether CSF PENK can serve not only as a mechanistically specific marker of striatal injury, but also as a practical tool for early disease monitoring and participant stratification in early‐stage clinical trials.

In conclusion, this study provides robust longitudinal evidence for CSF PENK as a predominantly striatum‐specific biomarker, in contrast to CSF NfL, which reflects widespread white matter change in far‐from‐onset HDGE cohorts. The strong association between lower baseline CSF PENK concentration and subsequent striatal gray and white matter volume reduction, together with its superior performance over NfL in discriminating between HD‐ISS stages 0 and 1, highlights its potential to enrich for clinical trials in HD‐ISS stage 0 and 1 cohorts. Conversely, the association between higher baseline CSF NfL concentration and longitudinal white matter reduction reinforces the role of NfL as an early marker of neuroaxonal injury in HD. Together, these findings demonstrate the complementary value of CSF PENK and NfL with imaging to detect and track the earliest pathological changes in HD, supporting their use in the design of future preventive trials.

## Author Roles

(1) Research Project: A. Conception, B. Organization, C. Execution; (2) Statistical Analysis: A. Design, B. Execution, C. Review and critique; (3) Manuscript: A. Writing of the first draft, B. Review and critique.

M.F.: 1A, 1B, 1C, 2A, 2B, 3A.

M.J.M.: 1B, 1C, 2C, 3B.

N.Z.H.: 1B, 1C, 2C, 3B.

M.L.: 1B, 1C, 2C, 3B.

K.F.: 1B, 1C, 2C, 3B.

O.T.: 1B, 1C, 2C, 3B.

J.G.: 1C, 2C, 3B.

M.C.: 1C, 2C, 3B.

A.H.: 1C, 2C, 3B.

H.Z.: 1C, 2C, 3B.

D.R.L.: 1C, 2C, 3B.

D.G.M.: 1C, 2C, 3B.

E.J.W.: 1A, 2C, 3B.

S.J.T.: 1A, 1B, 1C, 2C, 3B.

R.I.S.: 1A, 1B, 1C, 2A, 2B, 2C, 3B.

## Supporting information


**Table S1.** Characteristics of the total cross‐sectional voxel‐based morphometry (VBM) cohort (n = 149; 72 HD gene‐expanded [HDGE], 77 controls) used for group comparisons of grey and white matter volume.
**Table S2.** Characteristics of the total longitudinal voxel‐based morphometry (VBM) cohort (n = 88; 54 HD gene‐expanded [HDGE], 34 controls) used for group comparisons of grey and white matter volume change.
**Table S3.** Statistics for regional volumetric differences between HD gene‐expanded (HDGE) and control groups.
**Table S4.** Statistics for regional correlations between longitudinal grey and white matter volume change and baseline cerebrospinal fluid (CSF) proenkephalin (PENK) concentration in the HD gene‐expanded (HDGE) cohort (n = 50).
**Table S5.** Statistics for regional correlations between longitudinal grey and white matter volume change and baseline cerebrospinal fluid (CSF) neurofilament light (NfL) concentration in the HD gene‐expanded (HDGE) cohort (n = 50).
**Table S6.** Statistics for regional correlations between longitudinal grey and white matter volume change and baseline plasma neurofilament light (NfL) concentration in the HD gene‐expanded (HDGE) cohort (n = 50).

## Data Availability

De‐identified data may be made available 24 months following the completion of data collection, subject to review. Requests should be directed to the corresponding author. Approved applicants will be required to enter into a data‐sharing agreement with University College London (UCL).
