# Supplementary material for: Cerebrospinal Fluid Proenkephalin Predicts Striatal Atrophy Decades before Clinical Motor Diagnosis in Huntington's Disease
Source: Mov Disord. 2025 Sep 26;41(1):95–106. doi: 10.1002/mds.70062 (PMC12882043; doi:10.1002/mds.70062)
Supplement: Supplementary file 1 — Table S1. Characteristics of the total cross‐sectional voxel‐based morphometry (VBM) cohort (n = 149; 72 HD gene‐expanded [HDGE], 77 controls) used for group comparisons of grey and white matter volume. Table S2. Characteristics of the total longitudinal voxel‐based morphometry (VBM) cohort (n = 88; 54 HD gene‐expanded [HDGE], 34 controls) used for group comparisons of grey and white matter volume change. Table S3. Statistics for regional volumetric differences between HD gene‐expanded (HDGE) and control groups. Table S4. Statistics for regional correlations between longitudinal grey and white matter volume change and baseline cerebrospinal fluid (CSF) proenkephalin (PENK) concentration in the HD gene‐expanded (HDGE) cohort (n = 50). Table S5. Statistics for regional correlations between longitudinal grey and white matter volume change and baseline cerebrospinal fluid (CSF) neurofilament light (NfL) concentration in the HD gene‐expanded (HDGE) cohort (n = 50). Table S6. Statistics for regional correlations between longitudinal grey and white matter volume change and baseline plasma neurofilament light (NfL) concentration in the HD gene‐expanded (HDGE) cohort (n = 50). [file MDS-41-95-s001.docx]

**Supplementary Table 1:**

*Characteristics of the total cross-sectional VBM cohort (n=149; 72 HDGE, 77 controls) used for group comparisons of grey and white matter volume*

| **Characteristic** | **HDGE (n=72)** | **Controls (n=77)** | ***P value*** |
| --- | --- | --- | --- |
| Age at first scan (years) | 29.8 (5.5) | 29.9 (5.9) | 0.934 |
| Sex, n (%): Female:Male | 39:33 (54.2:45.8) | 43:34 (55.8:44.2) | 0.837 |
| HD-ISS (at time of scan) | N/A | N/A | N/A |
| Stage 0, n (%) | 48 (67) | N/A | N/A |
| Stage 1, n (%) | 23 (32) | N/A | N/A |
| Stage 2, n (%) | 1 (1) | N/A | N/A |
| MiSeq CAG repeat length | 42.2 (1.6) | N/A | N/A |
| CAP100 score at assessment | 55.5 (8.0) | N/A | N/A |
| DBS at assessment | 196.5 (36.5) | N/A | N/A |
| Estimated years to clinical motor diagnosis | 22.3 (5.2) | N/A | N/A |
| ICV (mL) | 1498.6 (152.6) | 1500.2 (140.2) | 0.945 |

*Values are n (%) or mean (SD), as appropriate. N/A indicates not applicable or not assessed in controls. P values are from two-sample t-tests (for continuous variables, e.g. age, ICV) or χ² tests (for categorical variables, e.g. sex).*

**Supplementary Table 2:**

*Characteristics of the total longitudinal VBM cohort (n=88; 54 HDGE, 34 controls) used for
group comparisons of grey and white matter volume change*

| **Characteristic** | **HDGE (n=54)** | | **Controls (n=34)** | ***P value*** |
| --- | --- | --- | --- | --- |
| Age at baseline (years) | 29.9 (5.6) | | 30.1 (5.9) | 0.839 |
| Sex, n (%): Female:Male | 26:26 (48.1:51.9) | | 20:14 (58.8:41.2) | 0.329 |
| Interval between visits (years) | 4.8 (0.6) | | 4.8 (0.6) | 0.738 |
| HD-ISS | Visit 1 | Visit 2 | N/A | N/A |
| Stage 0, n (%) | 44 (81) | 34 (63) | N/A | N/A |
| Stage 1, n (%) | 9 (17) | 19 (35) | N/A | N/A |
| Stage 2, n (%) | 1 (2) | 1 (2) | N/A | N/A |
| MiSeq CAG repeat length | 42.3 (1.5) | | N/A | N/A |
| CAP100 score at baseline | 55.8 (8.4) | | N/A | N/A |
| DBS at baseline | 198.1 (36.9) | | N/A | N/A |
| Estimated years to clinical motor diagnosis | 22.0 (5.2) | | N/A | N/A |

*Values are n (%) or mean (SD), as appropriate. N/A indicates not applicable or not assessed in controls. P values are from two-sample t-tests (for continuous variables, e.g. age, interval between visits) or χ² tests (for categorical variables, e.g. sex).*

**Supplementary Table 3:**

*Statistics for regional volumetric differences between HDGE and control groups*

| **Tissue type** | **Cluster size** | **Cluster regions** | **Peaks** | ***t*** | **FDR *q*** |
| --- | --- | --- | --- | --- | --- |
| Grey matter | 50299 | Bilateral striatum | Left putamen | 8.89 | 0.000 |
|  |  |  | Right putamen | 8.82 | 0.000 |
|  |  |  | Right putamen | 8.77 | 0.000 |
|  | 9443 | Right occipital lobe | Cuneus | 4.09 | 0.001 |
|  |  |  | Lingual gyrus | 3.93 | 0.002 |
|  |  |  | Lateral occipital gyrus | 3.88 | 0.003 |
|  | 7954 | Left occipital lobe | Lingual gyrus | 3.86 | 0.003 |
|  |  |  | Lateral gyrus | 3.85 | 0.003 |
|  |  |  | Lingual gyrus | 3.83 | 0.003 |
|  | 1199 | Left temporal lobe | Superior temporal gyrus | 3.43 | 0.010 |
| White matter | 18216 | Bilateral peristriatal | Left internal capsule | 9.71 | 0.000 |
|  |  |  | Right internal capsule | 8.55 | 0.000 |
|  |  |  | Right external capsule | 8.09 | 0.000 |
|  | 18411 | Right occipital lobe | Lateral occipital gyrus | 9.01 | 0.000 |
|  |  |  | Lateral occipital gyrus | 8.86 | 0.000 |
|  |  |  | Lateral occipital gyrus | 8.13 | 0.000 |

*Only clusters with > 1000 voxels are shown. Peaks represent up to three local maxima within the cluster region which are more than 8 mm apart. Statistics shown are at the peak level for a multiple regression model including age, sex, time interval and CAP100: t statistic and FDR q, significance after correction for multiple comparisons with the FDR.*

**Supplementary Table 4:**

*Statistics for regional correlations between longitudinal grey and white matter volume change and baseline CSF PENK concentration in the HDGE cohort (n=50)*

| **Tissue type** | **Cluster size** | **Cluster regions** | **Peaks** | ***t*** | **FDR *q*** |
| --- | --- | --- | --- | --- | --- |
| Grey matter | 11413 | Left striatum | Putamen | 4.46 | 0.013 |
|  |  |  | Putamen | 4.40 | 0.013 |
|  |  |  | Putamen | 4.32 | 0.013 |
|  | 13261 | Right striatum | Putamen | 4.45 | 0.013 |
|  |  |  | Putamen | 4.39 | 0.013 |
|  |  |  | Putamen | 4.38 | 0.013 |
|  | 1701 | Left occipital lobe | Lateral occipital gyrus | 4.36 | 0.013 |
|  | 5784 | Right occipital lobe | Lateral occipital gyrus | 4.27 | 0.013 |
|  |  |  | Lateral occipital gyrus | 4.24 | 0.013 |
|  |  |  | Lateral occipital gyrus | 4.08 | 0.013 |
|  | 2499 | Right temporal lobe | Inferior temporal gyrus | 4.10 | 0.013 |
|  |  |  | Inferior temporal gyrus | 3.97 | 0.013 |
|  |  |  | Inferior temporal gyrus | 3.59 | 0.020 |
|  | 1675 | Left occipital lobe | Pericalcarine gyrus | 4.06 | 0.013 |
| White matter | 14266 | Right peristriatal | Internal capsule | 5.10 | 0.008 |
|  |  |  | External capsule | 4.77 | 0.008 |
|  |  |  | Internal capsule | 4.43 | 0.008 |
|  | 15396 | Left peristriatal | Internal capsule | 4.73 | 0.008 |
|  |  |  | Internal capsule | 4.63 | 0.008 |
|  |  |  | Internal capsule | 4.27 | 0.008 |
|  | 4109 | Left occipital lobe | Lingual gyrus | 4.29 | 0.008 |
|  |  |  | Lateral gyrus | 4.02 | 0.008 |
|  |  |  | Cuneus | 4.00 | 0.009 |
|  | 1404 | Right temporal lobe | Inferior temporal gyrus | 3.85 | 0.010 |
|  |  |  | Inferior temporal gyrus | 3.43 | 0.020 |
|  |  |  | Inferior temporal gyrus | 3.34 | 0.023 |

*Only clusters with > 1000 voxels are shown. Peaks represent up to three local maxima within the cluster region which are more than 8 mm apart. Statistics shown are at the peak level for a multiple regression model including age, sex, time interval and CAP100: t statistic and FDR q, significance after correction for multiple comparisons with the FDR.*

**Supplementary Table 5:**

*Statistics for regional correlations between longitudinal grey and white matter volume change and baseline CSF NfL concentration in the HDGE cohort (n=50)*

| **Tissue type** | **Cluster size** | **Cluster regions** | **Peaks** | ***t*** | **FDR *q*** |
| --- | --- | --- | --- | --- | --- |
| Grey matter | 71954 | Bilateral occipital lobe | Lateral occipital gyrus | 7.03 | 0.000 |
|  |  |  | Cuneus | 6.34 | 0.000 |
|  |  |  | Lateral occipital gyrus | 5.71 | 0.000 |
|  | 1209 | Right parietal and temporal lobes | Postcentral gyrus | 4.96 | 0.001 |
|  |  |  | Postcentral gyrus | 3.35 | 0.017 |
|  |  |  | Postcentral gyrus | 2.90 | 0.038 |
|  | 4867 | Left parietal and temporal lobes | Insula | 4.78 | 0.001 |
|  |  |  | Parietal operculum | 3.87 | 0.006 |
|  |  |  | Temporal operculum | 3.84 | 0.007 |
|  | 3989 | Right frontal lobe | Inferior frontal gyrus | 4.62 | 0.002 |
|  |  |  | Inferior frontal gyrus | 3.88 | 0.006 |
|  |  |  | Inferior frontal gyrus | 3.61 | 0.011 |
|  | 4014 | Right striatum | Putamen | 3.60 | 0.011 |
|  |  |  | Caudate | 3.46 | 0.014 |
|  |  |  | Caudate | 3.20 | 0.022 |
|  | 1575 | Bilateral thalamus | Right thalamus | 3.40 | 0.016 |
|  |  |  | Left thalamus | 3.18 | 0.023 |
|  |  |  | Left thalamus | 3.03 | 0.030 |
| White matter | 351033 | Bilateral cerebral | Right lingual gyrus | 6.99 | 0.000 |
|  |  |  | Left lingual gyrus | 6.80 | 0.000 |
|  |  |  | Left postcentral gyrus | 6.63 | 0.000 |
|  | 5396 | Left cerebellum | Cerebellum | 2.81 | 0.009 |
|  |  |  | Cerebellum | 2.53 | 0.017 |
|  |  |  | Cerebellum | 2.34 | 0.025 |
|  | 5557 | Right cerebellum | Cerebellum | 2.71 | 0.012 |
|  |  |  | Cerebellum | 2.62 | 0.014 |
|  |  |  | Cerebellum | 2.43 | 0.021 |

*Only clusters with > 1000 voxels are shown. Peaks represent up to three local maxima within the cluster region which are more than 8 mm apart. Statistics shown are at the peak level for a multiple regression model including age, sex, time interval and CAP100: t statistic and FDR q, significance after correction for multiple comparisons with the FDR.*

**Supplementary Table 6:**

*Statistics for regional correlations between longitudinal grey and white matter volume change and baseline plasma NfL concentration in the HDGE cohort (n=50)*

| **Tissue type** | **Cluster size** | **Cluster regions** | **Peaks** | ***t*** | **FDR *q*** |
| --- | --- | --- | --- | --- | --- |
| Grey matter | 1013 | Right occipital lobe | Lateral occipital gyrus | 5.89 | 0.011 |
| White matter | 277380 | Bilateral cerebral | Left postcentral gyrus | 5.48 | 0.007 |
|  |  |  | Left parahippocampal gyrus | 5.38 | 0.007 |
|  |  |  | Left lingual gyrus | 5.17 | 0.007 |

*Only clusters with > 1000 voxels are shown. Peaks represent up to three local maxima within the cluster region which are more than 8 mm apart. Statistics shown are at the peak level for a multiple regression model including age, sex, time interval and CAP100: t statistic and FDR q, significance after correction for multiple comparisons with the FDR.*
